# Lobenzarit Attenuates DSS-Induced Colitis by Reprogramming Immune Microenvironment and Mitochondrial Homeostasis

**DOI:** 10.3390/ph19060926

**Published:** 2026-06-12

**Authors:** Ali Khaled, Manar A. Nader, Marwa E. Abdelmageed

**Affiliations:** 1 Department of Pharmacology and Toxicology, Faculty of Pharmacy, Mansoura University, Mansoura 35516, Egypt; dr.alikhaled87@yahoo.com (A.K.); manarahna@mans.edu.eg (M.A.N.); 2 Department of Pharmacology, Faculty of Pharmacy, Al-Nisour University, Baghdad 10001, Iraq; 3 Faculty Health Science Technology, Mansoura National University, Gamasa 7731168, Egypt

**Keywords:** dextran sulphate sodium, neutrophils infiltration, M1/M2 macrophages polarization, CD4+/IgM/IgE, TLR4/MAPK, tight junction

## Abstract

**Background:** The incidence of inflammatory bowel disease (IBD) is growing in the population. At present, the etiology of inflammatory bowel disease remains unclear, and there is no effective and low-toxic therapeutic drug. This study aimed to investigate the role of Lobenzarit (Lbz) in the treatment of colitis in mice as well as the underlying mechanism. **Methods:** In this experiment, colitis was induced in mice with dextran sulphate sodium (Dss). Subsequently, the role of Lbz in colitis and its underlying mechanisms were examined using H&E staining, TEM, ELISA, PCR, and other assays. **Results:** Lbz significantly attenuated the related symptoms of Dss-induced colitis in mice. In addition, Lbz suppressed neutrophil infiltration and restored macrophage polarization towards an anti-inflammatory state. Lbz also inhibited (*p* < 0.05) the activation of signaling pathways TLR4 and MAPK (51.61% decrease for TLR4 and 56.94% decrease for MAPK), reduced the release of inflammatory factors as it significantly decreased (*p* < 0.05) colonic IL-1β, TNF-α, IFN-γ, COX2, and VEGF (47.63, 42.49, 53.42, 58.74, and 61.28% decreases respectively) thereby attenuating the inflammatory response in mice. Lbz administration also restored the permeability of the intestinal barrier by increasing (*p* < 0.05) tight junction-associated proteins (claudin-1, occludin, and ZO-1 with a 5.36- and 2.26-fold increase for claudin-1 and ZO-1, respectively) and decreasing (*p* < 0.05) MALK levels by 53.51%. In addition, Lbz upregulated colonic Cytochrome C oxidase II, PDH, and ATP synthase levels and upregulated CD163, CD206, c-Maf, and PPAR-γ levels as compared to the DSS-treated group. **Conclusions**: Lbz has a repairing effect on Dss-induced colitis and may alleviate Dss-induced colitis by targeting the TLR4 pathway and promoting intestinal stem cell proliferation.

## 1. Introduction

Ulcerative colitis (UC) is a chronic, relapsing inflammatory illness of the bowel manifested by continuous inflammation of the colonic mucosa. UC typically starts in the rectum and extends proximally in a continuous way. UC is characterized by diminished epithelial barrier integrity, exaggerated immune responses, and persistent infiltration of inflammatory cells, developing symptoms such as diarrhea, rectal bleeding, abdominal pain, and weight loss [1,2].

Though the etiology of UC is yet unclear, it is extensively assumed that the disease develops from a complex interaction between genetic vulnerability, environmental elements, gut microbiota dysbiosis, and dysregulated immune reactions. Abnormal stimulation of innate and adaptive immunity plays a fundamental role, alongside enhanced generation of pro-inflammatory cytokines such as tumor necrosis factor- α (TNF-α), interleukin (IL)-1β, and IL-6, with the motivation of several signaling pathways comprising peroxisome proliferator-activated receptor gamma (PPARγ) and mitogen-activated protein kinases (MAPKs), that intensify intestinal inflammation and tissue damage [3,4].

Experimentally, dextran sulfate sodium (Dss)-provoked colitis in mice is one of the extremely utilized models of UC owing to its reproducibility and resemblance to human disease. Dss triggers epithelial barrier interruption, letting luminal antigens to infiltrate the mucosa and generate innate immune stimulation, causing oxidative stress and inflammatory cascade intensification [5].

Up-to-date therapeutic strategies for UC comprise corticosteroids, aminosalicylates, and immunosuppressive drugs. Nevertheless, their extended use is related to noteworthy adverse effects and an extreme rate of deterioration when withdrawn, emphasizing the need for safe and efficient immunomodulatory approaches [4].

Lobenzarit disodium (Lbz; commercially known as Kenzar or Arclon), also known as Disodium 4-chloro-2,2′-iminodibenzoate (CCA), is an immunomodulatory agent used for the treatment of autoimmune and chronic inflammatory diseases, predominantly rheumatoid arthritis and systemic lupus erythematosus. Lbz was historically established as a clinically effective disease-modifying antirheumatic drug (DMARD), widely recognized for its capacity to dampen chronic autoimmune responses by regulating T-cell and macrophage activities [6,7]. Lbz does not affect prostaglandin and leukotriene production, specifying a dissimilar mechanism of immune reaction modulation [8]. Lbz acts chiefly via adjustment of immune cell function, not direct inhibition of inflammatory mediators. It suppresses activated T- and B-lymphocyte responses [9,10,11], constrains autoantibody and immune complex development, and subsequently reduces the production of pro-inflammatory cytokines such as TNF-α and IL-1β [12,13], contributing to its disease-modifying effects in chronic inflammatory conditions. It also exhibited antioxidant properties [14], thus diminishing reactive oxygen species (ROS) and hence oxidative stress. In addition, it impedes constitutive nitric oxide (NO) and cyclic guanosine monophosphate (cGMP), providing further awareness of its molecular action as an immunomodulatory drug [15]. Due to its double immunomodulatory and antioxidant effects, Lbz has been assessed in various immune-mediated disorders as rheumatoid arthritis, systemic lupus erythematosus and autoimmune disorders with extreme lymphocyte triggering and autoantibody generation [7,16,17].

While Lbz clinical application was primarily focused on rheumatoid arthritis, research on this compound largely plateaued over the past few decades. However, the modern paradigm of drug repurposing offers a compelling rationale to revisit such an established immunomodulator [18,19,20]. Recent insights into inflammatory bowel diseases (IBDs), particularly ulcerative colitis, emphasize that chronicity is governed by an intimate crosstalk between mucosal mitochondrial failure and aberrant immune microenvironments [21]. Because the molecular pathways governing mitochondrial homeostasis and macrophage reprogramming were inaccessible during Lbz’s initial clinical era, its potential efficacy in mucosal inflammation remained completely overlooked. Crucially, Lbz has never been evaluated in experimental colitis. Therefore, this study aims to bridge this historical gap by evaluating, for the first time, whether Lbz can attenuate DSS-induced colitis, providing a novel therapeutic strategy through the dual action of immune microenvironment reprogramming and mitochondrial rescue.

## 2. Results

### 2.1. Lbz Attenuated Colitis in Dss-Treated Mice

**Figure 1:**
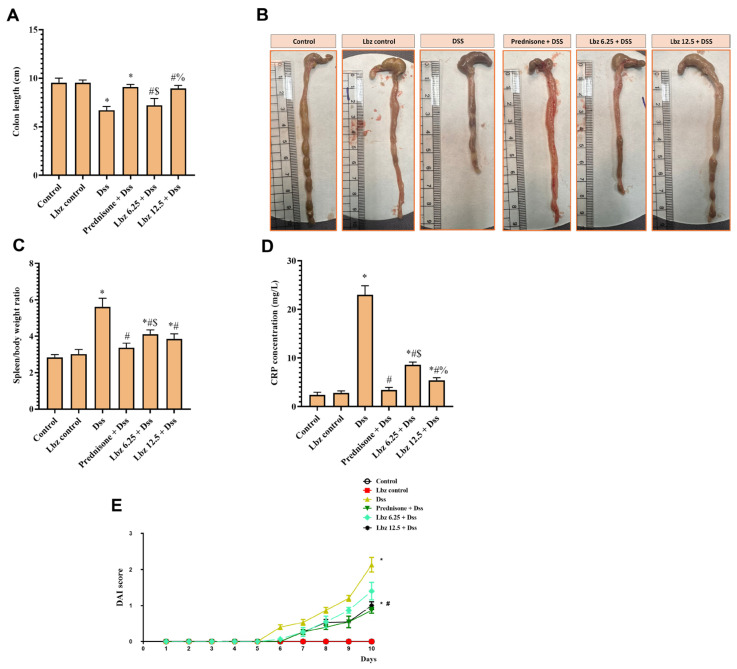
Effects of Lbz (6.25 and 12.5 mg/kg) and prednisone on colon length, spleen index, and CRP levels. (**A**) Colon length; (**B**) photographed colon tissues; (**C**) spleen index; (**D**) CRP levels; (**E**) DAI scores. Data are expressed as mean ± SD (*n* = 5). Dss: dextran sulphate sodium; CRP: C-reactive protein; DAI: disease activity index; Lbz: Lobenzarit. Statistically significant: * *p* < 0.05, compared to the control group; # *p* < 0.05, compared to the Dss group; $ *p* < 0.05 compared to the prednisone + Dss group; % *p* < 0.05 compared to the Lbz 6.25 + Dss group. Statistical analyses were performed using one-way ANOVA followed by the Tukey–Kramer multiple-comparisons post hoc test.

Compared with the control mice, the administration of Dss prompted a significant decrease in colon length (Figure 1A,B). On the other hand, the administration of Lbz 12.5 mg/kg and prednisone significantly reversed Dss-induced reduction in colon length compared with mice treated with Dss. It was also observed that Dss led to spleen enlargement and increased spleen index (Figure 1C). However, treatment with Lbz 12.5 mg/kg and prednisone was able to suppress Dss-induced spleen enlargement and decrease spleen index.

At the end of the treatments, Dss-induced colitis mice exhibited evident bloody diarrhea, and the DAI was calculated accordingly (Figure 1D). Elevated DAI scores were observed in Dss-treated mice but were significantly reduced following treatment with a high concentration of Lbz.

As shown in Figure 1E, compared to the control group, the production of serum CRP increased significantly in the Dss group. In contrast, serum CRP levels were significantly decreased in a dose-dependent manner following treatment with Lbz and prednisone compared to the Dss group. There was no significant difference between Lbz 12.5 + Dss and prednisone regarding serum CRP levels.

### 2.2. Lbz Action on the Structural and Ultrastructural Alteration in the Colon

**Figure 2:**
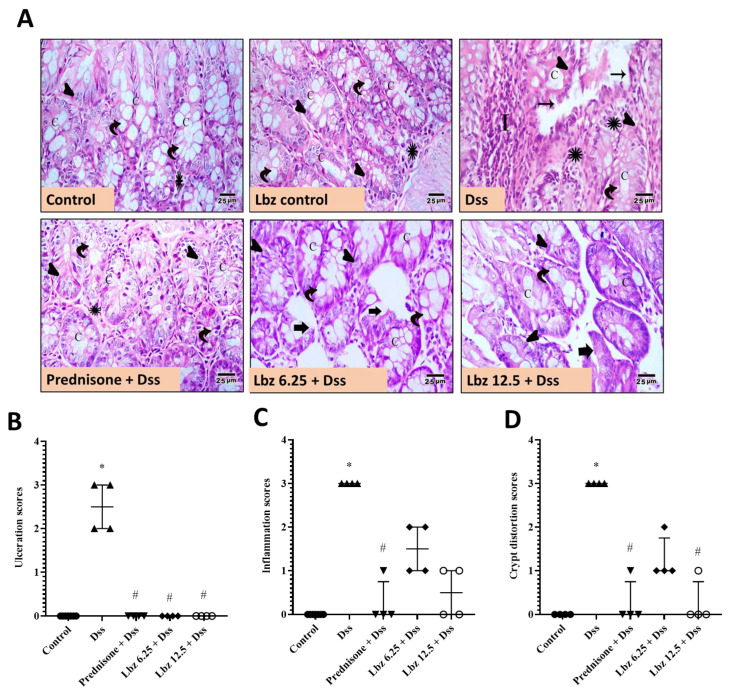
Effect of Lbz (6.25 and 12.5 mg/kg) and prednisone on Dss-induced histopathological changes in the colon of mice. (**A**) Photomicrographs of H&E-stained sections of the colon control group showed the mucosa formed of surface epithelium lined by columnar absorptive cells (arrowhead) and goblet cells (curved cells), lamina propria containing regularly arranged, tightly packed crypts (intestinal glands) (C). The simple columnar absorptive cells (arrowheads) have acidophilic cytoplasm and basal oval nuclei. Goblet cells (curved arrows) with foamy cytoplasm and basal flattened nuclei are also noticed. Numerous cells, most probably lymphocytes, are observed in the lamina propria (*). The Dss group showed loss of surface epithelium in some areas (thin arrows) with dark-stained nuclei of the columnar cells (arrowheads). Focal destructed disorganized mucosa with apparent decrease in goblet cells (curved arrows). Complete loss of the architecture (C) is observed. Heavy inflammatory cellular infiltration (I) is also observed. The lamina propria is infiltrated with inflammatory cells (*). Lbz control and prednisone groups are more or less similar to the control. Lbz 6.25 + Dss group showed some crypts (C) are lined with columnar absorptive cells (arrowhead) and goblet cells (curved arrows). These crypts are more or less similar to the control, while other crypts are irregular (thick arrows) and infiltrated by many inflammatory cells (*). Lbz 12.5 + Dss group showed most of the crypts are lined by columnar absorptive cells (arrowheads) and goblet cells (curved cells). A few focal destructed crypts are also seen with dark-stained nuclei of the columnar cells (thick arrows). (H&E X400). (**B**) Ulceration scores; (**C**) inflammation scores; (**D**) crypt distortion scores. Data were expressed as median ± interquartile range. Lbz: Lobenzarit; Dss: dextran sulphate sodium. Statistically significant: * *p* < 0.05, compared to the control group; # *p* < 0.05, compared to the Dss group, respectively, using Dunn’s multiple-comparison test following the Kruskal–Wallis test.

As shown in Figure 2, colonic photos from H&E-stained slices revealed the following: the control group showed the mucosa formed of surface epithelium lining by columnar absorptive cells and goblet cells, lamina propria containing regularly arranged, tightly packed crypts. The simple columnar absorptive cells have acidophilic cytoplasm and basal oval nuclei. Goblet cells with foamy cytoplasm and basal flattened nuclei were also noticed. Numerous cells, most probably lymphocytes, were observed in the lamina propria. Meanwhile, the Dss group showed loss of surface epithelium in some areas with dark-stained nuclei of the columnar cells, focal destruction, disorganized mucosa with apparent decrease in goblet cells. Complete loss of architecture and heavy inflammatory cellular infiltration were observed. The lamina propria was infiltrated by inflammatory cells. Meanwhile, the Lbz control and prednisone groups showed features comparable to those of the control group. The Lbz 6.25 + Dss group showed some crypts lined with columnar absorptive cells and goblet cells. These crypts are like the control mice, while other crypts are irregular and infiltrated by many inflammatory cells. The Lbz 12.5 + Dss group showed most of crypts lined by columnar absorptive cells and goblet cells. A few focally disrupted crypts were also observed, accompanied by darkly stained nuclei in the columnar cells.

**Figure 3:**
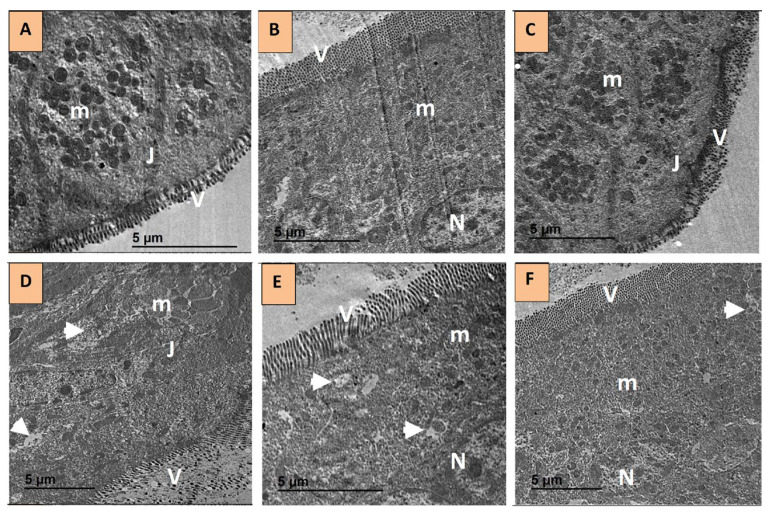
Effects of Lbz (6.25 and 12.5 mg/kg) and prednisone on Dss-induced histopathological changes using transmission electron micrographs (TEMs). The transmission electron micrographs of colonic tissue showed the following: (**A**) control group, with well-preserved epithelial ultrastructure, intact plasma membranes, densely arranged microvilli forming a continuous brush border, normal mitochondria, and preserved intercellular junctions; (**B**) the Lbz control group, which displayed ultrastructural features comparable to the control group. (**C**) The Dss group exhibited severe epithelial damage, characterized by disrupted and shortened microvilli, focal loss of the brush border, mitochondrial swelling with disrupted cristae, cytoplasmic vacuolization, and poorly defined junctional complexes. (**D**) Prednisone-treated group, showing marked attenuation of DDS-induced damage with restored microvillar organization, minimal mitochondrial swelling, reduced vacuolization, and largely preserved junctions. (**E**) Lbz 6.25 + Dss group demonstrated partial improvement, with focal microvillar restoration and moderate mitochondrial recovery, though the brush border remained discontinuous. (**F**) The Lbz 12.5 + Dss group, revealed dose-dependent ultrastructural improvement with substantial microvillar reorganization, preserved mitochondrial morphology, minimal vacuolization, and restored intercellular junctions, indicating enhanced mucosal barrier protection. Lbz: Lobenzarit; (m) mitochondrial; (N) nucleus; (J) junctions; (V) microvilli and (arrowhead) autophagosomes.

As shown in Figure 3, TEM photos of colonic tissue showed the control group with well-developed epithelial cells, uniformly oriented microvilli forming a dense and continuous apical border, elongated and structurally intact mitochondria, sharply delineated junctional complexes, indicating a competent epithelial barrier and rare or absent autophagosomes (Figure 3A). The Lbz-alone group exhibited epithelial cells with microvilli, mitochondrial morphology, and junctional organization comparable to the control group (Figure 3B). Meanwhile, the Dss group revealed marked ultrastructural derangements, characterized by severe microvillar blunting and fragmentation with extensive loss of apical organization, swollen and rounded mitochondria, poorly defined or partially detached junctional complexes, reflecting barrier breakdown, and numerous autophagosomes and autophagic vacuoles within the cytoplasm (Figure 3C). On the other hand, the prednisone-treated group showed pronounced re-establishment of microvillar architecture with restoration of apical polarity, largely normalized mitochondrial morphology, and mostly reassembled junctional complexes, supporting improved epithelial cohesion (Figure 3D). The Lbz 6.25-treated group demonstrated focal improvement in microvillar arrangement, although apical continuity remained incomplete, partial mitochondrial recovery, intermittently recognizable junctional complexes, and scattered autophagosomes (Figure 3E). The Lbz 12.5-treated group induced marked dose-dependent recovery, evidenced by substantial reorganization of microvilli and near-normal apical alignment, predominantly intact mitochondria, clearly re-established junctional complexes, and sparse autophagosomes (Figure 3F).

### 2.3. Lbz Impact on the Integrity of the Intestinal Barrier

**Figure 4:**
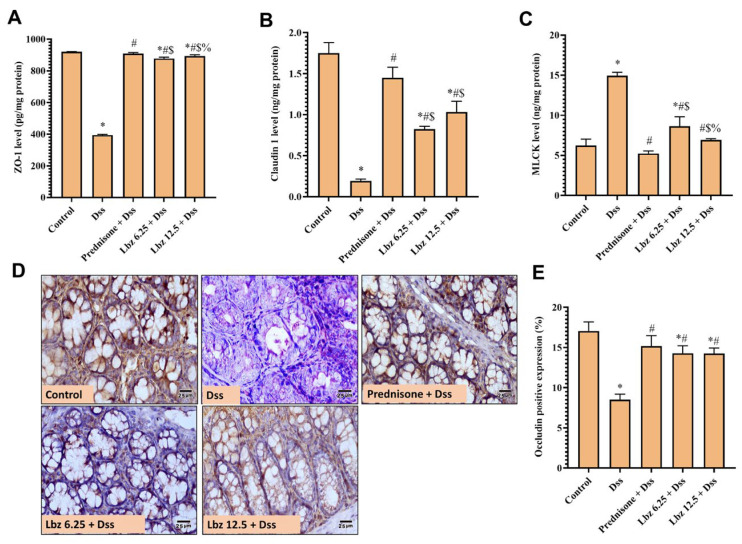
The effect of Lbz (6.25 and 12.5 mg/kg) and prednisone on colonic levels of ZO-1, claudin-1, MALK, and occludin. (**A**) ZO-1 level; (**B**) claudin-1 level; (**C**) MLCK level; (**D**) occludin immune figure; (**E**) bar graph showing the % expression of occludin. Expression in the cytoplasm of the cells (thick arrows) and the lamina propria (thin arrows). Data are expressed as mean ± SD (*n* = 4). Lbz: Lobenzarit; Dss: dextran sulphate sodium; MALK: ATP-hydrolyzing subunit of the maltose/trehalose transport system; ZO-1: Zonula Occludens-1. Statistically significant: * *p* < 0.05, compared to the control group; # *p* < 0.05, compared to the Dss group; $ *p* < 0.05 compared to the prednisone + Dss group; % *p* < 0.05 compared to the Lbz 6.25 + Dss group. Analyses were performed using one-way ANOVA followed by the Tukey–Kramer multiple-comparisons post hoc test.

Disruption of intestinal barrier integrity in Dss-treated mice was associated with reduced ZO-1 and claudin-1 levels (Figure 4A,B) and elevated MLCK (Figure 4C) levels compared to the control group. The administration of Lbz and prednisone greatly restored all previously measured biomarkers compared to the Dss-treated mice. The dose-dependent effects of Lbz on all these biomarkers are taken into account. There is no significant difference between Lbz 12.5 + Dss and prednisone in most of those biomarkers.

The protein expression levels of occludin were further examined to confirm damaged intestinal barrier by Dss. Photomicrographs of occludin-immunostained sections of the colon (Figure 4D) revealed marked positive brown signals of occludin on the membrane and in the cytoplasm of the control group. On the other hand, Dss mice showed that the intensity of brown signals was weaker than that in the control group. The prednisone + Dss group exhibited features similar to those of the control group and showed no significant differences compared to the control mice. The Lbz 6.25 + Dss group showed moderate brown signals on the membrane and in the cytoplasm. The Lbz 12.5 + Dss group showed strong brown signals on the membrane and in the cytoplasm although less intense than those of the control group. The % positive protein expression of occludin in the colon tissue of the Dss group was markedly lower than that of the control group. Compared with the Dss group, the % area expression of occludin protein in the Lbz and prednisone groups increased significantly. Lbz at both doses produced no significant changes compared with prednisone (Figure 4E).

### 2.4. Lbz Effect on Colonic Immune Response (CD4+, IgM, and IgE)

**Figure 5:**
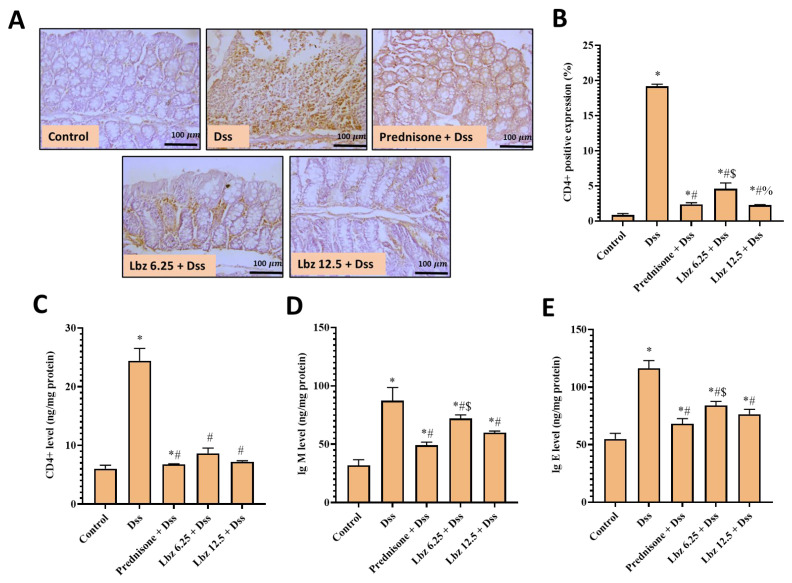
Effects of Lbz (6.25 and 12.5 mg/kg) and prednisone on colonic levels of CD4+, IgM, and IgE: (**A**) CD4+ immune figure (image magnification= 100×: Bar 100 μm); (**B**) bar graph showing the % expression of CD4+; (**C**) CD4+ level; (**D**) IgM level (**E**) IgE level. Data are expressed as mean ± SD (*n* = 4). Lbz: Lobenzarit; Dss: dextran sulphate sodium; CD4+: cluster of differentiation 4; IgM: immunoglobulin M; IgE: immunoglobulin E. Statistically significant: * *p* < 0.05, compared to control group; # *p* < 0.05, compared to the Dss group; $ *p* < 0.05 compared to the prednisone + Dss group; % *p* < 0.05 compared to the Lbz 6.25 + Dss group. Analyses were performed using a one-way ANOVA, followed by the Tukey–Kramer multiple-comparisons post hoc test.

Representative IHC of CD4+ expression and localization in the colonic sections of different treatment groups showed that the control group exhibited few to mild immunopositive-stained lamina propria lymphocytes, whereas the DSS-treated group showed diffuse, high-intensity immunopositive-stained infiltrating lymphocytes within the inflamed area. On the other hand, the prednisone group showed scattered faintly stained lymphocytes. The Lbz 6.25 pretreated group showed scattered multifocal immunopositive stained laminapropria infiltrating CD4+ T cells, and the Lbz 12.5 pretreated group showed mild immunostained laminapropria lymphocytes (Figure 5A,B). Additionally, Dss greatly enhanced T and B lymphocyte recruitment, as proven by a marked increase in CD4+, IgM, and IgE protein content (Figure 5C–E) compared to the control group. Both the Lbz intervention and the positive control groups markedly normalized T and B lymphocytes-mediated response. There was a dose-dependent effect between the two Lbz doses, with no significant difference between Lbz 12.5 + Dss and prednisone in most measured parameters.

### 2.5. Lbz Effect on Colonic Immune Cell Infiltration

**Figure 6:**
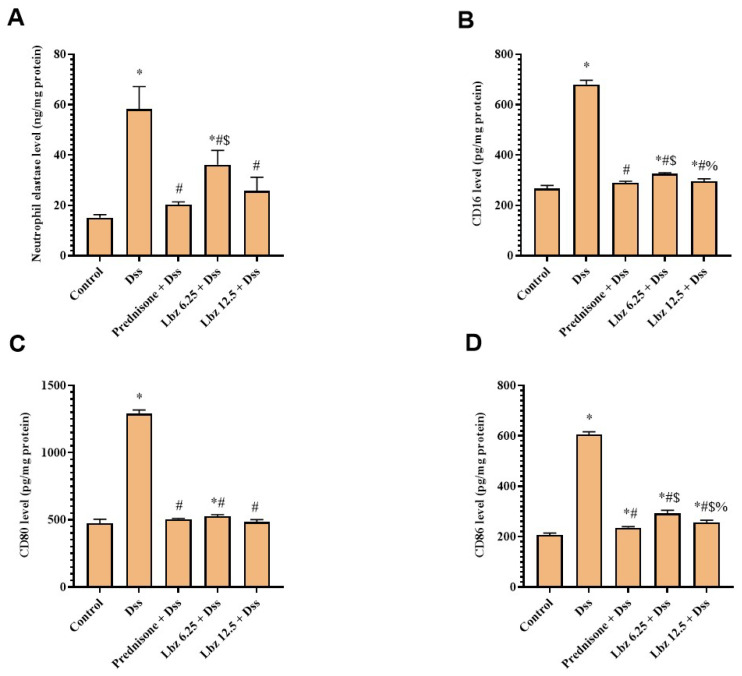
Effects of Lbz (6.25 and 12.5 mg/kg) and prednisone on neutrophil infiltration and M1/M2 macrophage polarization in the colon of Dss-induced colitis. (**A**) neutrophil elastase; (**B**) CD16; (**C**) CD80; (**D**) CD86. Data are expressed as mean ± SD (*n* = 4). Lbz: Lobenzarit; Dss: dextran sulphate sodium; CD: cluster of differentiation. Statistically significant: * *p* < 0.05, compared to the control group; # *p* < 0.05, compared to the Dss group; $ *p* < 0.05 compared to the prednisone + Dss group; % *p* < 0.05 compared to the Lbz 6.25 + Dss group. All analyses were performed using a one-way ANOVA, followed by the Tukey–Kramer multiple-comparisons post hoc test.

Elevated neutrophils infiltration upon Dss installation is evident by elevating the colonic levels of neutrophil elastase and CD16 (Figure 6A,B). This was followed by colonic M1/M2 macrophage polarization toward an inflammatory state, as proved by marked elevation in the protein content of CD80 and CD86; M1 macrophage activation (Figure 6C,D) and diminution of CD163 (protein expression) (Figure 6A,B), CD206, and c-MAF; M2 macrophage deactivation (Figure 6C,D). Both the Lbz intervention and the positive control groups markedly suppressed neutrophil elastase and CD16, proving reduced neutrophil infiltration and reduced CD80 and CD86, with elevated CD163, CD206, and C-MAF, indicating macrophage polarization towards an anti-inflammatory state. There was a dose-dependent effect between the two Lbz doses, with no significant difference between Lbz 12.5 + Dss and prednisone in most measured parameters.

**Figure 7:**
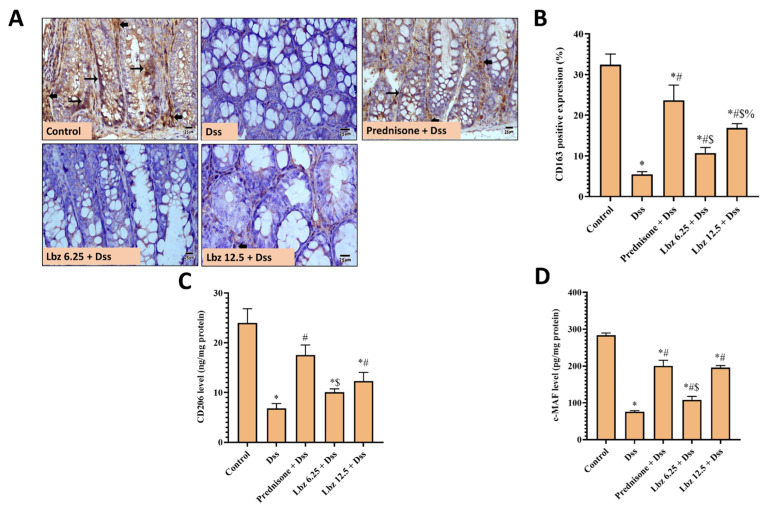
Effects of Lbz (6.25 and 12.5 mg/kg) and prednisone on colonic expression levels of CD163, CD206, and c-Maf. (**A**) CD163 immune figure; (**B**) bar graph showing the % expression of CD163; (**C**) CD206 level; (**D**) c-Maf level. Expression in the cytoplasm of the cells (thick arrows) and the lamina propria (thin arrows). Data are expressed as mean ± SD (*n* = 4). Dss: dextran sulphate sodium; CD: cluster of differentiation; c-Maf: Musculoaponeurotic Fibrosarcoma Oncogene Homolog. Statistically significant: * *p* < 0.05, compared to the control group; # *p* < 0.05, compared to the Dss group; $ *p* < 0.05, compared to the prednisone + Dss group; % *p* < 0.05, compared to the Lbz 6.25 + Dss group. Analyses were performed using one-way ANOVA, followed by the Tukey–Kramer multiple-comparisons post hoc test.

To further confirm macrophage polarization CD163 immunostaining expression (Figure 7A) was measured. Photomicrographs of CD163 immunostained sections of the colon of the control group showed the marked expression in both the cytoplasm of cells and the lamina propria. On the other hand, the Dss group showed diminishing expression of CD163 in the cytoplasm of the cells and the lamina propria. While the prednisone positive control + Dss group exhibited features similar to those of the control group. Additionally, the Lbz 6.25 + Dss group showed moderate immune expression in the cytoplasm of the cells, and the lamina propria and Lbz 12.5 + Dss group showed high immune expression in the lamina propria. The Dss-administered mice displayed a lower percentage of colonic CD163-positive cells than healthy mice. Following Lbz intervention at both doses and prednisone as a positive control, colonic CD163 positive protein expression was dramatically elevated compared to Dss mice. Notably, there was a dose-dependent effect between the two Lbz doses (Figure 7B).

### 2.6. Lbz Impact on the Colonic Inflammatory Cascade

**Figure 8:**
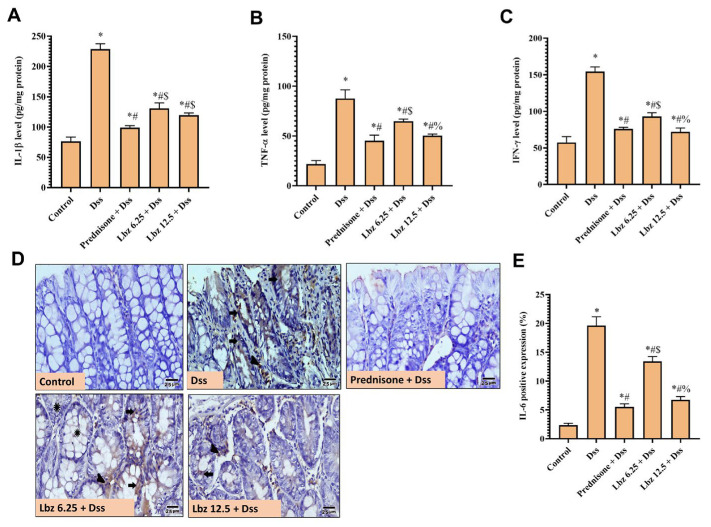
Effects of Lbz (6.25 and 12.5 mg/kg) and prednisone on colonic levels of pro-inflammatory cytokines in the colon of Dss-induced colitis. (**A**) IL-1β level; (**B**) TNF-α level; (**C**) IFN-γ level; (**D**) IL-6 immune figure; (**E**) bar graph showing the % expression of IL-6. Expression in the cytoplasm of the cells (thick arrows) and the lamina propria (thin arrows). Data are expressed as mean ± SD (*n* = 4). Lbz: Lobenzarit; Dss: dextran sulphate sodium; IL-1β: interleukin-1 beta; TNF-α: tumor necrosis factor-alpha; IFN-γ level: interferon-gamma. Statistically significant: * *p* < 0.05, compared to the control group; # *p* < 0.05, compared to the Dss group; $ *p* < 0.05, compared to the prednisone + Dss group; % *p* < 0.05 compared to the Lbz 6.25 + Dss group. Analyses were performed using one-way ANOVA, followed by the Tukey–Kramer multiple-comparisons post hoc test.

**Figure 9:**
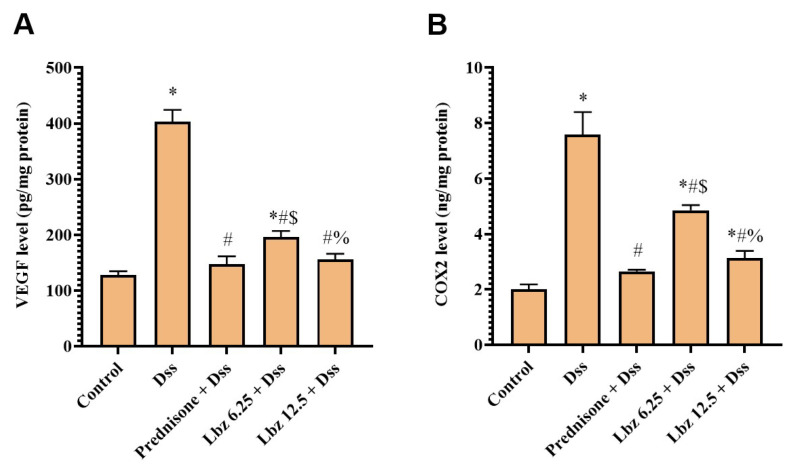
Effects of Lbz (6.25 and 12.5 mg/kg) and prednisone on colonic levels of VEGF and COX2. (**A**) VEGF level; (**B**) COX2 level. Data are expressed as mean ± SD (*n* = 4). Lbz: Lobenzarit; Dss: dextran sulphate sodium; COX2: hepatic levels of cyclooxygenase-2; VEGF: Vascular Endothelial Growth Factor. Statistically significant: * *p* < 0.05, compared to the control group; # *p* < 0.05, compared to the Dss group; $ *p* < 0.05, compared to the prednisone + Dss group; % *p* < 0.05, compared to the Lbz 6.25 + Dss group. Analyses were performed using one-way ANOVA, followed by the Tukey–Kramer multiple-comparisons post hoc test.

As shown in Figure 8, the Dss administration dramatically increased the colonic levels of IL-1β, TNF-α, and IFN-γ (Figure 8A–C) as a consequence of immune cell activation, with subsequent elevation of VEGF levels, protein expression, and COX2 (Figure 9) in comparison to the control mice, indicating a powerful shift toward inflammation. Interestingly, low and high doses of Lbz intervention and prednisone significantly restored all these biomarkers compared to the Dss mice. Dose-related effects from both doses of Lbz, with the high dose showing an insignificant effect from St prednisone in most measured parameters.

IL-6 was further measured by IHC (Figure 8D). Photomicrographs of IL-6-immunostained sections of the colon of the control group showed the negative immunostained expression in both the cytoplasm of cells and the lamina propria. Meanwhile, the Dss group showed a marked expression in the cytoplasm of the cells and the lamina propria. On the other hand, the prednisone + Dss group was comparable to the control group. In addition, the Lbz 6.25 + Dss group showed moderate immune expression in the cytoplasm of the cells, and the lamina propria and other crypts showed negative expression in both the lamina propria and the cytoplasm of cells. The Lbz 12.5 + Dss group showed few immune expressions in the lamina propria and in the cytoplasm of cells. The colonic positive protein expression percentage of IL-6 (Figure 8E) was significantly increased in the Dss mice compared with that in healthy mice. After Lbz intervention by both doses and prednisone, the colonic positive expression percentage of IL-6 was dramatically decreased in mice with colitis. Notably, there was a dose-dependent effect between the two Lbz doses, with no significant difference between Lbz12.5 + Dss and prednisone.

### 2.7. Lbz Impact on Colonic Molecular Pathway (TLR4, MAPK p38, JNK, PI3K, p-Akt, PPAP-γ and ERK2)

**Figure 10:**
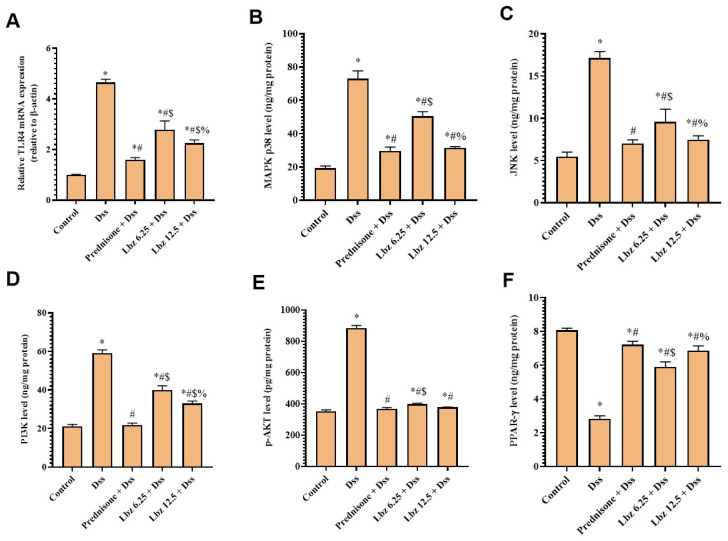
Effects of Lbz (6.25 and 12.5 mg/kg) and prednisone on intracellular signaling pathways targeting inflammation in the colon of Dss-induced colitis. (**A**) Relative quantitation of TLR4 mRNA expression (fold change relative to β-Actin); (**B**) MAPK p38; (**C**) JNK; (**D**) PI3K; (**E**) p-Akt; (**F**) PPAR-γ. Data are expressed as mean ± SD (*n* = 4). Lbz: Lobenzarit; Dss: dextran sulphate sodium; TLR4: Toll-like Receptor 4; MAPK p38: mitogen-activated protein kinase p38; JNK: c-Jun N-terminal Kinase; PI3K: Phosphatidylinositol 3-Kinase; p-Akt: phosphorylated protein kinase B; PPAR-γ: peroxisome proliferator-activated receptor gamma. Statistically significant: * *p* < 0.05, compared to control group; # *p* < 0.05, compared to the Dss group; $ *p* < 0.05, compared to the prednisone + Dss group; % *p* < 0.05, compared to the Lbz 6.25 + Dss group, Analyses were performed using one-way ANOVA, followed by Tukey–Kramer multiple-comparisons post hoc test.

As revealed in Figure 10, Dss administration dramatically increased the colonic TLR4 mRNA expression and MAPK p38, JNK, PI3K, and p-Akt protein levels (Figure 10A–E) in the colitis mice compared to the control mice. Interestingly, the low and high dose of Lbz intervention and prednisone significantly restored all these biomarkers compared to the Dss mice. Dose-related effects were observed with both doses of Lbz, with the high dose showing an insignificant effect from prednisone in most of those parameters. Furthermore, the level of colonic PPAR-γ (Figure 10F) of the Dss-treated group was significantly lower than that of the mice in the control group. However, treatment with low and high doses of Lbz, as well as prednisone pretreatment significantly elevated the level of colonic PPAR-γ in mice with colitis compared to Dss mice. Notably, there was a dose-dependent effect between the two Lbz doses, with no significant difference between Lbz 12.5 + Dss and St prednisone.

**Figure 11:**
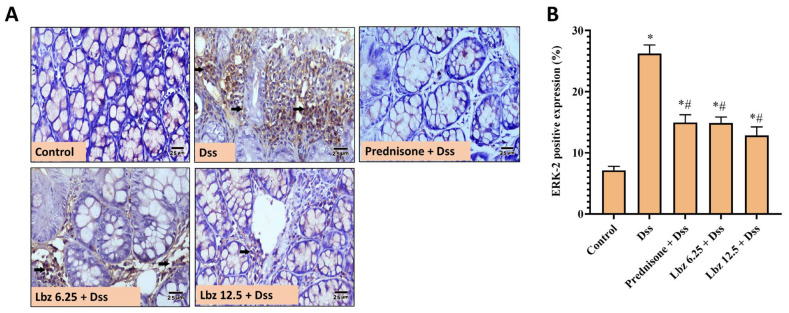
Effects of Lbz (6.25 and 12.5 mg/kg) and prednisone on colonic expression levels of ERK2. (**A**) ERK2 immune figure; (**B**) bar graph showing the % expression of ERK2 in the cytoplasm of the cells (thick arrows) and the lamina propria (thin arrows). Data are expressed as mean ± SD (*n* = 4). Lbz: Lobenzarit; Dss: dextran sulphate sodium; ERK2: Extracellular Signal-Regulated Kinase 2. Statistically significant: * *p* < 0.05, compared to the control group; # *p* < 0.05, compared to the Dss group compared to the Lbz 6.25 + Dss group. Analyses were performed using one-way ANOVA, followed by the Tukey–Kramer multiple-comparisons post hoc test.

Photomicrographs of ERK2 immunostained sections (Figure 11A) of the colon of the control group showed the negative immunostained protein expression in cells of the lamina propria. Meanwhile, the Dss group showed strong brown expression of ERK2 in the cells of the lamina propria. On the other hand, the prednisone + Dss group was similar to the control group. The Lbz 6.25 + Dss group showed moderate brown staining in the cells of the lamina propria. In addition, the Lbz 12.5 + Dss group showed weak brown staining in the cells of the lamina propria. The percentage of ERK2-positive cells in the colon was significantly increased in the Dss-treated mice compared with healthy mice. Following treatment with Lbz at both doses and prednisone, the colonic positive protein expression percentage of ERK2 was dramatically decreased in the mice with colitis (Figure 11B).

### 2.8. Lbz Effect on Colonic Oxidant/Antioxidant Balance

**Figure 12:**
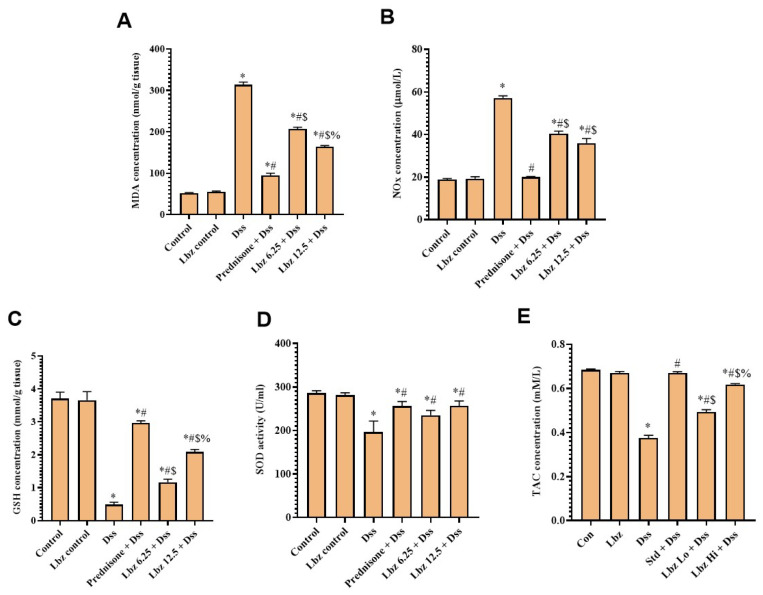
Effects of Lbz (6.25 and 12.5 mg/kg) and prednisone on colonic levels of colonic oxidative stress and antioxidant biomarkers. (**A**) MDA; (**B**) Nox; (**C**) SOD; (**D**) GSH; (**E**) TAC. Data are expressed as mean ± SD (*n* = 5). Lbz: Lobenzarit; GSH: reduced glutathione; TAC: total antioxidant capacity; SOD: superoxide dismutase; MDA: malondialdehyde; NOx: nitrite. Statistically significant: * *p* < 0.05, compared to the control group; # *p* < 0.05, compared to the Dss group; $ *p* < 0.05, compared to the prednisone + Dss group; % *p* < 0.05 compared to the Lbz 6.25 + Dss group. All analyses were performed using one-way ANOVA, followed by the Tukey–Kramer multiple-comparisons post hoc test.

Mice administered Dss exhibited marked imbalance in oxidant/antioxidant status reflected by noteworthy elevation in MDA/NOx colonic levels with diminution of GSH, SOD, and TAC contents compared with the control group (Figure 12A–E), while administration of prednisone and Lbz in Dss mice restored this imbalance compared to Dss mice. There was a dose-dependent effect concerning Lbz intervention, with the high dose exhibiting a more pronounced effect than the low dose regarding colonic MDA and TAC levels.

### 2.9. The Effect of Lbz on Colonic Mitochondrial Energy Metabolism

**Figure 13:**
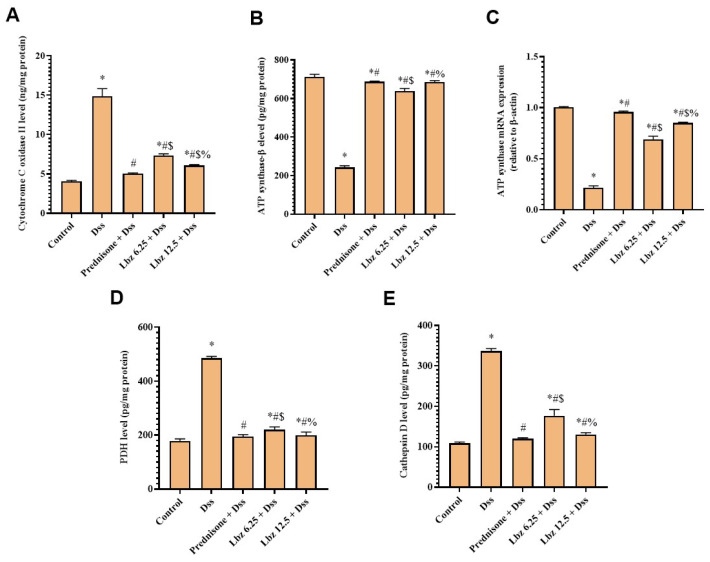
Effects of Lbz (6.25 and 12.5 mg/kg) and prednisone on the mitochondrial energy metabolism and the integrity of the intestinal barrier of Dss-induced colitis. (**A**) Cytochrome C oxidase II level; (**B**) PDH level; (**C**) ATP synthase level; (**D**) relative quantitation of ATP synthase mRNA expression (fold change relative to β-Actin); (**E**) Cathepsin D level. Data are expressed as mean ± SD (*n* = 4). Lbz: Lobenzarit; ATP: adenosine triphosphate; Dss: dextran sulphate sodium; PDH: Pyruvate Dehydrogenase. Statistically significant: * *p* < 0.05, compared to the control group; # *p* < 0.05, compared to the Dss group; $ *p* < 0.05, compared to the prednisone + Dss group; % *p* < 0.05, compared to the Lbz 6.25 + Dss group. Analyses were performed using one-way ANOVA, followed by the Tukey–Kramer multiple-comparisons post hoc test.

The administration of Dss, as depicted in Figure 13, led to a significant exhaustion of mitochondrial energy, reflected by an increase in the colonic cytochrome C oxidase II levels (Figure 13A), along with a decrease in ATP synthase protein content and mRNA expression (Figure 13B,C). This mitochondrial energy damage was accompanied by a metabolic shift toward glycolysis, reflected by elevated PHD (Figure 13D) and deterioration of the mitochondrial–lysosomal axis, as reflected by elevated Cathepsin D (Figure 13E) compared to the control group. The administration of Lbz at both doses and prednisone greatly restored all previously measured biomarkers compared to the Dss mice. The fact that the high dose of Lbz demonstrated superior effects compared with the low dose of Lbz for all those biomarkers, with no significant difference between Lbz 12.5 + Dss and prednisone for most biomarkers, should be taken into consideration.

## 3. Discussion

The current study demonstrated that Lbz exerts a significant dose-dependent protective effect against Dss-induced UC in mice. While Lbz did not fully reverse the systemic signs such as weight loss and colon shortening, it profoundly modulated the molecular architecture and inflammatory signaling within the colonic tissue.

The primary pathological feature of Dss-induced colitis is the disruption of the intestinal physical barrier. Our results showed that Lbz significantly mitigated the loss of occludin, ZO-1, and claudin-1. This is critical because the loss of these TJ proteins facilitates the “leaky gut” syndrome. Additionally, the observed elevation in MLCK in the Dss group, a known trigger for tight junction (TJ) dysregulation, is reduced by Lbz in the peri-junctional actomyosin ring, thereby maintaining barrier competence [22,23]. This was visually confirmed by our TEM findings, where Lbz-treated mice exhibited reorganized microvilli and restored junctional complexes.

A pivotal finding in this study is the ability of Lbz to restore the levels of CD4+ T lymphocytes, IgM, and IgE to near-normal values in mice with Dss-induced colitis. The pathogenesis of UC involves a complex interplay between innate and adaptive immunity, where the breakdown of the mucosal barrier leads to an aberrant systemic immune response.

Prominently, this harmonized immune modulation spreads to humoral immunity. The witnessed lessening in IgG, IgE, and IgM levels can be deduced as a downstream consequence of repressed cytokine signaling, predominantly IL-6 and IL-1β, which are critical drivers of B-cell activation, differentiation, and class switching. Furthermore, the weakening of CD4+ T-cell stimulation limits B-cell help, thus leading to diminished immunoglobulin generation.

CD4+ infiltration suggests an interruption of the adaptive immune phase. In our Dss model, the marked increase in colonic CD4+ infiltration signifies the recruitment of helper T cells to the site of injury, which further amplifies the inflammatory cascade through the secretion of pro-inflammatory cytokines such as TNF-α and IFN-γ [3]. The administration of Lbz effectively reversed this infiltration. As an immunomodulator, Lbz has been shown to inhibit the proliferative response of T cells and their subsequent migration to inflamed tissues [14]. By normalizing CD4+ levels, Lbz likely limits the “fuel” for chronic inflammation, preventing the progression from acute mucosal damage to systemic immune activation.

The restoration of humoral immune response (IgM and IgE) levels to baseline in our Dss mice upon use of Lbz is perhaps one of the most significant results of this intervention. The elevation of IgM in colitis reflects a systemic response to the translocation of commensal bacteria across the “leaky” gut barrier. By restoring IgM to normal levels, Lbz indicates dual success: first, repair of the physical barrier (as confirmed by our TEM and TJ protein data), and second, direct stabilization of B-cell hyperactivation [24]. Additionally, the normalization of IgE is particularly striking. In Dss models, elevated IgE is often associated with mast cell degranulation and type I hypersensitivity-like reactions within the mucosa, which further increases intestinal permeability [3]. The ability of Lbz to bring IgE back to normal levels suggests that it interrupts the mast cell–IgE axis, thereby reducing mucosal edema and hypersensitivity to luminal antigens.

In addition to evaluating CD4+ cells and immunoglobulin profiles (IgM/IgE), this study characterized the recruitment and phenotypic changes of neutrophils and macrophages within the colonic mucosa. The significant reduction in neutrophile elastase levels in the Lbz-treated groups is a critical indicator of decreased neutrophilic infiltration. Neutrophils are among the first responders in Dss-induced colitis, releasing neutrophil elastase and ROS, which exacerbate tissue damage. By suppressing neutrophile elastase, Lbz limits the acute proteolytic destruction of the extracellular matrix [25]. This aligns with the reduction in CD16 (NK cells/neutrophils), suggesting that Lbz dampens the early innate immune activation signal.

Our results showed a significant decrease in CD163/CD206/c-Maf (M2-like/regulatory marker) and elevated CD80/CD86 (M1-like/activation markers) in Dss mice. In the context of colitis, excessive recruitment of both phenotypes can contribute to a “cytokine storm [11]. By modulating M1/M2 polarization toward anti-inflammatory status, Lbz helps “reset” the macrophage microenvironment. From previous results, it is obvious that restoration of CD4+, IgG, and IgE levels was closely mirrored by a significant attenuation in macrophage polarization towards anti-inflammatory status and neutrophilic recruitment, indicating a comprehensive suppression of both innate and adaptive immune branches and humoral response by Lbz.

The witnessed downregulation of TLR4 expression employs a vital regulatory action on both innate and adaptive immune reactions. As a key pattern-recognition receptor, TLR4 activation presents a serious upstream incident for leukocyte recruitment and activation within the lamina propria. By limiting the “gateway” receptor (TLR4) and subsequent PI3K/Akt signaling, Lbz restricts the activation and infiltration of neutrophils and macrophages, as well as the expansion of CD4+ T cells, thus diminishing the global inflammatory burden [26]. Lbz ensures that even the cells that do reach the lamina propria remain in a less activated, less destructive state.

Mechanistically, TLR4 signaling orchestrates downstream activation of MAPKs, including p38, JNK, and ERK, which are vital for translating extracellular stress signals into transcriptional activation of pro-inflammatory mediators [3]. Preservation of this axis by Lbz diminishes cytokine generation and interrupts the positive feedback loop that endures leukocyte activation and tissue infiltration.

Concurrently, the repression of TLR4/MAPK signaling fundamentally alters macrophage functional behavior. This attenuated cascade coordinates a decisive transition away from the destructive M1 polarization state toward a more controlled, homeostatic profile, thereby dampening the local generation of crucial inflammatory cytokines. This action is additionally strengthened by the reestablishment of PPAR-γ, which acts as a molecular brake on NF-κB signaling, eventually diminishing transcriptional programs linked to T-cell stimulation and macrophage-mediated inflammation [26,27].

Mutually, these outcomes come to suppress key inflammatory and angiogenic mediators, including TNF-α, IL-1β, IL-6, COX2, VEGF, and IFN-γ. By targeting numerous interrelated signaling hubs, including TLR4, MAPKs, and NF-κB, while restoring PPAR-γ activity, Lbz efficiently interrupts the crosstalk between immune cell infiltration, macrophage polarization, and antibody-mediated responses, resulting in a broad reduction of intestinal inflammation.

In UC, the colonic mucosa is displayed to distinct oxidative stress, which seems to be firmly related to mitochondrial dysfunction and cellular metabolic imbalance [28,29,30,31]. In the current work, Lbz evidently improved antioxidant protections, as supported by elevated GSH, SOD, and TAC levels, besides reducing MDA and Nox levels. These findings suggest that Lbz exerts a dual defensive action by both scavenging excess ROS/RNS and strengthening the endogenous antioxidant defense.

Notably, the diminution of oxidative stress was complemented by an obvious enhancement in mitochondrial function [28,29,30]. Dss-induced variations, comprising diminished ATP synthase expression and raised cytochrome c oxidase II and PDH levels, reveal a state of mitochondrial metabolic stress where colonocytes struggle to withstand energy homeostasis. Lbz efficiently restored ATP synthase expression, demonstrating a potential role in mitochondrial metabolic reprogramming and recovery of cellular bioenergetics.

Furthermore, the interaction between oxidative and mitochondrial stress was further supported by the modulation of autophagy-related markers. The observed reduction in Cathepsin D expression, along with the decreased formation of autophagosomes in TEM, suggests that Lbz moderates unnecessary autophagic flux and inhibits lysosomal membrane permeabilization, a processes often prompted by mitochondrial damage and oxidative overload and closely associated with epithelial programmed cell death [31,32].

While current biological therapies, such as anti-TNF agents (e.g., Infliximab) and JAK inhibitors (e.g., Tofacitinib), have revolutionized the management of ulcerative colitis, they are often associated with high costs and primary or secondary non-responsiveness in a significant subset of patients. Lbz, as a small molecule immunomodulator, offers a different mechanistic approach by simultaneously targeting mitochondrial bioenergetics and a broad spectrum of immune cells (CD4+, CD80+, CD163+).

Limitations of this study: Despite the potent anti-inflammatory and mucosal protective effects observed in this study, several limitations must be acknowledged. First, the clinical use of Lbz has been limited by reports of nephrotoxicity (e.g., proteinuria) and gastrointestinal intolerance. Although not assessed in our short-term model, long-term safety remains a key concern. Additionally, systemic administration may cause off-target effects; thus, colon-targeted delivery or structural optimization is needed to preserve efficacy while reducing renal exposure. Moreover, the Dss model reflects acute injury but not the chronic, relapsing course of UC; therefore, validation in chronic or spontaneous colitis models is required. Second, although ELISA assays for CD antigens were successfully performed, these data alone are not sufficient to conclusively demonstrate macrophage invasion or polarization. Flow cytometry (FACS) analysis would provide more definitive evidence; however, due to practical constraints, this approach could not be implemented within the current study. Therefore, the interpretation of our findings has been cautiously limited to suggesting a potential impact on processes such as macrophage invasion and polarization, rather than definitive mechanistic conclusions.

## 4. Materials and Methods

### 4.1. Animals

Healthy adult male BALB/C mice (weighing 20–25 g) were purchased from the Egyptian Organization for Biological Products and Vaccines (VACSERA), Giza, Egypt. Animals were housed 5 per cage. There was free access to food and water. Animals were kept in constant environmental conditions. The temperature was constant at 25 ± 2 °C, with regular light cycles of 12/12–10/14 h light/dark with 60–70% humidity. The maintenance protocol of animals was approved by the “Animal Care and Use Committee”, Mansoura University, Egypt, under approval code No. MU-ACUC (PHARM.MS.24.10.116), dated 20 October 2024, in compliance with ethical and reporting standards of the ARRIVE guidelines.

### 4.2. Reagents

Lbz disodium (Lbz) was purchased from Macklin Biochemical Co., Ltd., Shanghai, China (Cat No. 64808-48-6). It was dissolved in normal saline (0.9% NaCl) for intraperitoneal (IP) administration. Dss (molecular weight 36,000–50,000 Da) was purchased from RSI Reagent Science Industry Limited, Cat No. 9011-18-1, Lot. 11032-258, Hong Kong, (China), and it was dissolved in drinking water. Other materials used were of analytical grade.

### 4.3. Experimental Design

**Figure 14:**
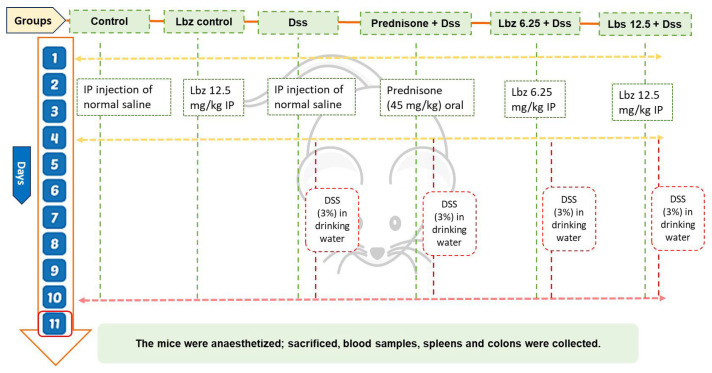
Schematic presentation of the experimental design. Dss: dextran sulphate sodium; IP: intraperitoneal; Lbz: Lobenzarit.

To induce experimental colitis, we replaced the standard drinking water of the mice with 3% DSS for 7 consecutive days, spanning from day 4 to day 10 [33,34]. Lbz was administered intraperitoneally (IP) once daily for 10 days at doses of either 6.25 or 12.5 mg/kg body weight. These specific experimental windows were chosen as sub-therapeutic fractions calculated from historically documented mouse benchmarks. Specifically, we scaled down from baseline ranges of 2–10 mg/kg, administered orally in chronic autoimmune frameworks [7], and 25–100 mg/kg IP, applied in acute tissue injury protocols [35,36,37]. This sub-therapeutic dosing rationale was mathematically structured and cross-verified against modern translational pharmacology frameworks and standard FDA animal-to-human dose conversion guidelines [38,39]. This approach ensures that our dosing matrix sits safely below established systemic toxicological thresholds while retaining its targeted microenvironmental immunomodulatory potential. To ensure translational relevance, the mouse doses were converted to human equivalent doses (HEDs) using the FDA-recommended body surface area (BSA) method [38]. A dose of 6.25 mg/kg in mice corresponds to ~30 mg/day in a 60 kg adult, while 12.5 mg/kg corresponds to ~61 mg/day. These values represent approximately 12.5–25% of the clinically used oral dose of lbz in rheumatoid arthritis (120–240 mg/day), confirming that our regimens were deliberately chosen as sub-therapeutic fractions. In DSS-induced colitis, oral drug absorption is severely compromised due to mucosal destruction and diarrhea. Therefore, IP administration was selected to ensure reproducible systemic exposure. Importantly, IP absorption occurs via the portal circulation, preserving hepatic first-pass metabolism similar to oral dosing, while bypassing the disrupted gastrointestinal tract. Additionally, IP delivery facilitates direct exposure to peritoneal immune compartments, enhancing drug access to macrophages and T cells that traffic to the inflamed colon. By employing IP administration of sub-therapeutic fractions, we achieved consistent systemic bioavailability and targeted mucosal protection. Thirty male BALB-C mice were randomly divided into 6 groups (5 mice in each group) (Figure 14):

Group I (control group): mice received a single IP injection of normal saline daily for 10 days.

Group II (Lbz control group): mice received a single IP injection of Lbz (12.5 mg/kg) daily for 10 days.

Group III (Dss group): mice received a single IP injection of normal saline daily for 10 days and Dss solution from day 4 to day 10.

Group IV (Lbz 6.25 + Dss group): mice were administered Lbz (6.25 mg/kg, IP, daily) for 10 days and Dss solution from day 4 to day 10.

Group V (Lbz 12.5 + Dss group): mice were administered Lbz (12.5 mg/kg, IP, daily) for 10 days and Dss solution from day 4 to day 10.

Group VI (prednisone + Dss group) served as the positive control group. Mice received prednisone (45 mg/kg) orally [40] for 10 days and Dss solution in drinking water from day 4 to day 10. The body weight, stool characteristics, and blood in the stool were monitored daily for each mouse. The disease activity index (DAI) was assessed using a previously established method [41].

After the completion of the experimental period and on the 11th day of investigation, all mice were fasted for 4 h, weighed, and anesthetized with thiopental sodium (70 mg/kg, IP), and then, blood samples were collected from the heart, left to clot for 30 min, and then centrifuged at 3000× *g* at 4 °C for a further 15 min to get serum for biochemical measurements. Then, the colon and spleen were harvested, cleaned with cold phosphate-buffered saline (PBS), and dried with tissue paper. The spleen was weighed to measure the spleen index (spleen weight/body weight × 100).

The separated colon was quickly flushed with PBS using a 5–10 mL syringe fitted with an 18G-3” straight feeding needle (2.25 mm ball) to remove feces and blood, photographed, and then divided into 4 parts. One part was fixed with 4% paraformaldehyde and used for hematoxylin–eosin (HE) staining and immunohistochemical (IHC) analysis. Another part was immersed in 2.5% glutaraldehyde for Transmission Electron Microscopy (TEM) examination. The third part was homogenized in PBS, yielding 10% *w*/*v* homogenates for the detection of oxidative stress indicators and ELISA measurements. The fourth part was flash-frozen in liquid nitrogen and stored at −80 °C for further molecular analyses.

### 4.4. Estimation of C-Reactive Protein and Oxidative Stress Biomarkers

Serum C-reactive protein (CRP) levels were determined using Spinreact (Barcelona, Spain) according to the manufacturer’s instructions. Colon tissue homogenates were used for the measurement of malondialdehyde (MDA) and total antioxidant capacity (TAC) levels, which were detected using commercially available kits (Biodiagnostic kits, Cairo, Egypt, Cat. No. MD25 29 and TA25 13, respectively), according to the manufacturer’s protocols. Additionally, levels of nitrate/nitrite (NOx; cat no. NO 25 33), reduced glutathione (GSH; cat no. GR 25 11), and superoxide dismutase (SOD; cat no. SD 25 21) were measured in colon tissue homogenates using commercial assay kits obtained from Bio-Diagnostic Company, Giza, Egypt, according to the manufacturer’s instructions.

### 4.5. Histopathological Examination

Colon samples were dehydrated in increasing alcohol grades, cleaned with xylene, and then embedded in paraffin wax. The embedded samples were cut at a thickness of 5 μm using a microtome. The section was stained with hematoxylin and eosin (H&E) and then examined under the light microscope (Olympus CH2, Olympus, Tokyo, Japan) [42]. Multiparametric scoring was used to assess the histopathological changes in colon sections of different treatment groups. The assessed parameters are the extent of mucosal ulceration, the severity of inflammation, and the degree of crypt distortion.

### 4.6. TEM Analysis

Colon tissue samples were fixed in 2.5% glutaraldehyde, post-fixed in osmium tetroxide, dehydrated in graded ethanol series, and embedded in epoxy resin. Ultrathin sections were stained with uranyl acetate and lead citrate, then examined using a transmission electron microscope to evaluate mitochondrial morphology and ultrastructural epithelial changes. Using a specialized razor, the colonic tissues were cut into pieces for fixation, then washed with PBS solution and fixed in 1% osmium tetrachloride [43,44]. Sections were prepared at the Electron Microscopy Unit, Mansoura University. An electron microscope JEOL_JEM-2100 (Jeol, Tokyo, Japan) was used to take pictures of the colon tissue. Due to technical and economic constraints, TEM findings were reported descriptively based on independent qualitative assessment by two pathologists. Image processing and analysis were conducted using Digital Micrograph and Soft Imaging Viewer software (Gatan Microscopy Suite, version 2.11.1404.0).

### 4.7. ELISA Assessment

The colonic levels of the following markers were measured using commercially available mouse ELISA kits in accordance with the manufacturer’s instructions (Table 1).

### 4.8. IHC Analysis

Colon tissue sections (4–5 µm) were deparaffinized in xylene and rehydrated through a graded ethanol concentration. Antigen retrieval was done utilizing citrate buffer (10 mM, pH 6.0) for 10 min in a microwave oven, then cooled at room temperature. Endogenous peroxidase activity was blocked with 3% hydrogen peroxide for 10 min. Non-specific binding was minimized by incubation with 5% bovine serum albumin (BSA) at room temperature for 30 min.

The following antibodies were used: CD4 (cat no. 25-0042-82, dilution 1:100), occludin (cat no. 33-1500, dilution 1:200), CD163 (cat no. 14-1631-82, dilution 1:100), ERK2 (cat no. MA1-099, dilution 1:200), and IL-6 (cat no. 701028, dilution 1:100), all purchased from Thermo Fisher Scientific Anatomical Pathology (46360 Fremont Blvd., CA 94538, USA). These antibodies were utilized for the biotin–avidin complex method [45] and for IHC analysis of CD4, occludin, CD163, ERK2, and IL-6 in colonic tissues. The colonic sections were then incubated with primary antibodies at 4 °C overnight. After washing, slides were incubated with biotinylated secondary antibody (1:200) for 30 min, then with streptavidin-HRP conjugate at room temperature for 30 min. Visualization was achieved using 3,3′-diaminobenzidine (DAB) substrate, and counterstaining was performed with hematoxylin. Slides were examined using a trinocular microscope (MBL4000-T-F-LED- Krüss Optronic GmbH, Hamburg, Germany). The percentage of positive areas was investigated in 4 slices per group via image J analysis software version 1.54 (FIJI, National Institutes of Health, Bethesda, MD, USA). The average percentage was computed.

### 4.9. Quantitative Real-Time PCR

Total RNA was extracted from colon tissue homogenate using the SV Total RNA Isolation System (Thermo Scientific, Waltham, MA, USA), following the supplier’s instructions, where colon tissues were homogenized in lysis buffer, and RNA was bound to silica membrane columns, washed to remove contaminants, and eluted in RNase-free water. The purity and concentration of RNA were assessed spectrophotometrically at 260/280 nm, and integrity was verified by agarose gel electrophoresis. cDNA synthesis was performed using a reverse transcription kit (Cat No. K4374966, Thermo Fisher Scientific, New York, NY, USA) that was used to reverse-transcribe one microgram of RNA from each sample into complementary DNA (cDNA), as indicated by the manufacturer’s instructions. Genomic DNA contamination was eliminated by the DNase treatment included in the kit protocol. Reverse transcription was done under the recommended thermal cycling conditions, and the obtained cDNA was then stored at −20° until use. Real-time qPCR amplification and analysis were performed using an Applied Biosystem with software version 3.1 (StepOne™, Panama City, FL, USA). Gene expressions of TLR-4 and ATP synthase were quantified using real-time PCR and normalized to housekeeping gene beta (β-) actin. Relative gene expression was calculated using the 2^−ΔΔCt^ method. The used primers are listed in Table 2.

### 4.10. Statistical Analysis

A priori sample size was determined based on previous studies and power analysis (power = 0.8, α = 0.05) to detect biologically relevant differences between groups. Each experimental group included a minimum of five independent biological replicates (*n* ≥ 4). Parameters that showed normal distribution were used as a one-way analysis of variance (ANOVA). Tukey’s multiple-comparison post-test was used in case of significance between groups, and the data were expressed as mean ± standard deviation (SD). Data were analyzed by the Shapiro–Wilk normality test for normality. Nevertheless, data that did not show a normal distribution were analyzed using nonparametric tests such as Kruskal–Wallis tests, and the data were expressed as median and interquartile range. Statistical analysis was performed by GraphPad Prism 9.0 (GraphPad Software Inc., San Diego, CA, USA). A statistical probability of *p* value < 0.05 was considered statistically significant.

## 5. Conclusions

Current data revealed that Lbz affords a multi-target protection action which imitates several effects of modern biologics, such as anti-TNF-α and anti-IFN-γ, with the extra benefit of enhancing PPAR-γ and direct mitochondrial protection. Taken together, our results highlight Lbz as a promising candidate for UC management, with potential roles in both induction and maintenance of remission. The current experimental design largely represents a maintenance paradigm due to its prophylactic approach. Therefore, future studies should be directed toward assessing its therapeutic efficacy in remission induction under active disease conditions. From a clinical trial perspective Phase I studies should establish safety, tolerability, and pharmacokinetics in healthy volunteers. Phase II trials are needed to assess efficacy in patients with active UC, with emphasis on remission induction and biomarker validation (mitochondrial function, oxidative stress). Phase III trials should compare Lbz with standard biologic therapies, evaluating long-term remission maintenance, relapse prevention, and safety outcomes. Given its multi-target mechanism, Lbz may be positioned as an adjunct or alternative therapy for patients refractory to conventional biologics, particularly those with mitochondrial failure-driven disease phenotypes.

## Figures and Tables

**Table 1:** List of ELISA kits used in this study.

Marker	Cat no.	Company, Origin
Cluster Of Differentiation 4 (CD4)	MBS2506108	MyBiosource, San Diego, CA, USA
Myosin Light Chain Kinase (MLCK)	MBS260887	
Zonula Occludens-1 (ZO-1)	MBS706128	
Claudin-1	MBS724224	
Cluster of Differentiation 16 (CD16)	MBS1756428	
CD80	MBS825050	
CD206	MBS2606199	
Interleukin-1 Beta (IL-1β)	MBS175967	
Tumor Necrosis Factor Alpha (TNF-α)	MBS825075	
Cyclooxygenase-2 (COX2)	MBS720812	
Phosphoinositide 3-Kinase (PI3K)	MBS162296	
Peroxisome Proliferator-Activated Receptor Gamma (PPAR-γ)	ELK1633	ELK biotechnology, Sugar Land, TX 77478, USA
Phosphorylated Protein Kinase B (p-Akt)	ELK0791	
c-Jun N-terminal Kinase (JNK)	ELK0946	
Cytochrome c Oxidase	ELK7630	
Pyruvate Dehydrogenase (PDH)	LS-F4366	LSBio, Newark, CA 94560, USA
Cluster of Differentiation 86 (CD86)	LS-F15288	
p38 Mitogen-Activated Protein Kinase (MAPK p38)	MOFI00965	AssayGennie, Dublin, Ireland
MAF bZIP Transcription Factor (c-Maf)	MOEB0980	
ATP Synthase, Complex V (ATP synthase II)	MOEB1082	
Interferon Gamma (IFN-γ)	CSB E04578m	Cusbio, Houston, TX, USA
Neutrophil Elastase	E-EL-M3025	Elabscience, Houston, TX, USA
Vascular Endothelial Growth Factor (VEGF)	E-EL-M1292	
Immunoglobulin E (IgE)	E-EL-M3107	
Immunoglobulin M (IgM)	E-EL-M3036	
Cathepsin D (Cathepsin family proteases)	NBP2-67258	Novus Biologicals, Centennial, CO, USA

**Table 2:** Primers sequences used for RT-PCR.

Primer	Sequence		Source
TLR4	Forward	AGCTTCTCCAATTTTTCAGAACTTC	GENE BANK ACCN: N M_021297.1
	Reverse	TGAGAGGTGGTGTAAGCCATGC	
ATP synthase	Forward	TGGTGAAGAGACTGACGGATGC	GENE BANK ACCN: NM_007505.1
	Reverse	TCAAAGCGTGCTTGCCGTTGTC	
Beta actin	Forward	CATTGCTGACAGGATGCAGAAGG	GENE BANK ACCN: NM_007393.1
	Reverse	TGCTGGAAGGTGGACAGTGAGG	

## Data Availability

The data presented in this study are available on request from the corresponding author. (the data are not publicly available due to privacy or ethical restrictions.)
